# Unusual Displacement of a Mobilised Dental Bridge during Orotracheal Intubation

**DOI:** 10.1155/2011/781957

**Published:** 2011-10-20

**Authors:** Paolo Feltracco, Stefania Barbieri, Francesca Salvaterra, Letizia Tiano, Rosa Maria Gaudio, Helmut Galligioni, Carlo Ori, Francesco Maria Avato

**Affiliations:** ^1^Dipartimento di Farmacologia e Anestesia, Clinica di Anestesia e Medicina Intensiva, Università degli Studi di Padova, 35100 Padova, Italy; ^2^Dipartimento di Scienze Biomediche e Terapie Avanzate, Sezione di Medicina Legale, Università degli Studi di Ferrara, 44121 Ferrara, Italy

## Abstract

*Dental trauma during tracheal intubation mostly happens in case of poor dentition, restricted mouth opening, and/or difficult laryngoscopy.* 57-year-old man undergoing laparoscopic radiofrequency ablation of unresectable hepatocellular carcinoma had his dental work detached at induction of anesthesia. Oropharyngeal direct view, manual inspection, fibreoptic nosendoscopy, tracheobronchoscopy, and fiberoptic inspection of the esophagus and stomach were unsuccessful in locating the dislodged bridge. While other possible exams were considered, such as lateral and AP x-ray of head and neck, further meticulous manual “sweepings” of the mouth were performed, and by moving the first and second fingers below the soft palate deep towards the posterolateral wall of the pharynx, feeling consistent with a dental prosthesis was detected in the right pharyngeal recess. Only after pulling the palatopharyngeal arch upward was it possible to grasp it and extract it out with the aid of a Magill Catheter Forceps. Even though the preexisting root and bridge deficits were well reported by the consultant dentist, the patient was fully reimbursed. The lack of appropriate documentation of the advanced periodontal disease in the anesthesia records, no mention of potential risks on anesthesia consent, and insufficient protective measures during airway instrumentation reinforced the reimbursement claim.

## 1. Introduction

In recent years, significant interest has been addressed to dental trauma during anaesthesia procedures. Dental injury mostly happens during tracheal intubation, and it is more frequent in case of poor dentition and/or difficult laryngoscopy as occurs in case of restricted mouth opening, macroglossia, retrognathism, prominent incisors, shortened thyromental distance, limited neck extension, and so forth. Emergency intubation and poor visualization of the glottis along with a low training level of the anesthesiologist performing the intubation are among the factors which determine the incidence and the type of dental injury [1]. Even in skilled hands, the upper maxillary incisors are the most frequently damaged teeth when some difficulty is encountered and considerable force is applied. Damage to native teeth or artificial prostheses is often associated with patient complaints and represents up to one third of potential or confirmed malpractice claims against anesthesiologists [2]. 

## 2. Case Report

A 57-year-old man underwent laparoscopic radiofrequency ablation of unresectable hepatocellular carcinoma under general anesthesia. He was on a waiting list for liver transplantation because of hepatitis-C-related end-stage liver failure. Signs of portal hypertension, malnutrition, and a severe restrictive pattern on spirometry were the significant findings of his preoperative clinical status. At preanesthesia evaluation, the patient referred that he had had a fixed dental prosthesis for many years. He reported no trouble masticating or any irregular motility of the dental arch. This three-element fixed bridge of metal alloy with resin front beat was made of two crowns on the natural roots of the left lateral incisor and right central incisor. On oral inspection, the patient was classified as Mallampati II; no predictive signs of difficult intubation were reported. Other specific conditions of dental work were not documented in the anesthesiological chart. General anesthesia was induced with propofol and a nondepolarizing muscle relaxant. In order to maintain satisfactory blood oxygenation, face mask ventilation with Guedel cannula was performed before tracheal intubation. Due to the reduced lung compliance, tight adherence of face mask and high manual compression pressure on the reservoir bag were necessary to guarantee an adequate inspiratory flow. Repeated forceful manual insufflations were applied before laryngoscopy. Laryngoscopic inspection and glottis view were not as easy as expected; as a consequence, tracheal intubation was not immediate. While leveraging the laryngoscope against the upper dental work, the artificial prosthesis detached from its position and dropped into the oral cavity. It was not seen during tracheal tube placement; however, the laryngoscopic view was quite restricted. Immediately after securing the airway, the visible parts of the oral cavity were accurately inspected but the bridge was not found. The digital exploration which followed was also fruitless.

**Figure 1:**
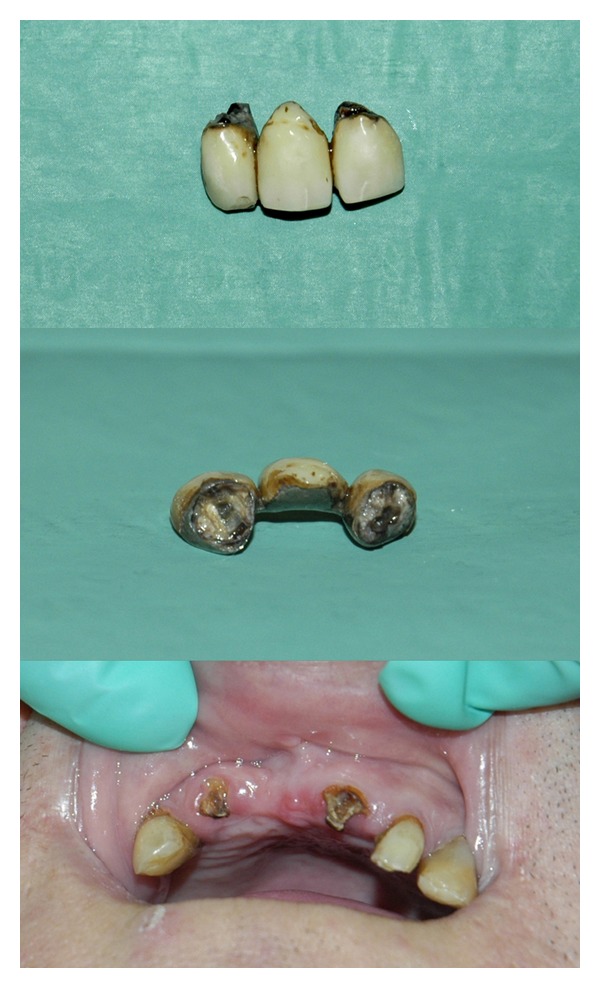
Detached dental bridge and teeth decay under the prosthesis.

To exclude the possibility that the bridge had slipped into the tracheobronchial tree, a tracheobronchoscopy was performed but nothing was found in the main airways. The next step consisted in direct visualization of the nasopharyngeal opening and the oropharynx by a fiber-optic nose endoscopy via both nostrils, but the prosthesis was still not found. A subsequent fiberoptic inspection of the esophagus and the stomach was also unsuccessful in locating the bridge. The upper abdomen X-ray, performed next in order to exclude a hidden dislocation within the stomach, was also futile. While other possible exams were considered, such as lateral and AP X-ray of head and neck, further meticulous manual “sweepings” of the mouth were performed, and by moving the first and second fingers below the soft palate deep towards the posterolateral wall of the pharynx, feeling consistent with a dental prosthesis was detected in the right pharyngeal recess. Multiple unsuccessful attempts at digital removal of the prosthesis followed. Only after pulling the palatopharyngeal arch upward was it possible to grasp it and extract it out with the aid of a Magill Catheter Introducing Forceps. At the emergence from anesthesia, the patient did not complain of soreness of the gums but he did complain about the lost prosthesis, as was expected. Before discharge from the recovery room, he was informed of the way the damage occurred and was offered a dental consult. The consulting dentist noted a complete avulsion at root portion (Figure 1) and provided a brief description of the dental lesion and detached dental work. 

Both the anesthesiologist's and the dentist's reports were included in the incident reporting record which was sent to the Legal Medicine Department and then to the Hospital Insurance Company. After “processing” the clinical case, a few days after discharge, our patient received a formal letter from the Hospital Insurance stating that the full cost of a new bridge would have been covered. 

## 3. Discussion

 Dental lesions are frequent complications of orotracheal intubation, and a wide variety of factors are responsible for this. In the case reported, at preoperative evaluation, the oral visual field was not described as restricted, and no frank weakness or major defects of the prosthesis were noted. This is the reason no particular precautions were taken and no mouthguards were applied. 

According to the anesthesiologist's report, even though visualization of the glottis was reduced, laryngoscopy was neither impetuous nor aggressive; nevertheless, an involuntary attempt at using the bridge as a fulcrum for the laryngoscope blade was enough to displace the artificial dental work. It is very likely that the bridge had already been partly moved by previous forceful manual mask ventilation which was required to ventilate the restrictive lung parenchyma. The force transmitted by the flat reinforced bite portion of the Guedel cannula prevalently on the bridge likely caused an injury that was not discovered at manual opening of the mouth but was significant enough to dislodge the prosthesis at first contact with the laryngoscope. Once it dislodged and had fallen into the oral cavity, the relatively large bridge (Figure 1) was not visible during intubation nor was it discovered at subsequent mouth inspection. Neither the bronchoscopy nor the esophagogastroendoscopy had identified a possible inadvertent aspiration or an unrecognized ingestion. Surprisingly indeed it was not detected by the bilateral nose-fiberoptic inspection of pharyngeal cavity either. 

It is likely that the edematous swollen mucosa of the nose, pharynx, mouth, and so forth, typical features of end stage cirrhosis, could have impeded the direct visualization of the dental work once it had moved laterally into the pharyngeal recess. Only a repeated deep posterolateral digital exploration could detect the “hidden” bridge thanks to its particular consistency to the touch.

The unrecognized weakness of dental roots favoured the detachment of the prosthesis. Chronic deficit of protein synthesis predisposes cirrhotic patients to gingival atrophy, periodontal disease, chronic inflammation, and bacterial infection. These processes lead to dissolution of periodontium and weakness of dental roots. 

The dentist's inspection of the fractured roots demonstrated remarkable tooth decay (Figure 1) which had already worn down the steadiness of the prosthesis thus preventing the possibility of future repair. 

The bridge showed a break between the metal and the abutment, which had allowed the infiltration of oral bacteria and consequent progression of tooth decay under the prosthesis. 

At this state, even a minimal trauma with the Guedel cannula was sufficient to move the prosthesis [3]. Light force by the laryngoscope blade likely completed the damage.

Knowledge of a possible roots weakness could have helped avoid recurring to the Guedel airway [4]. Impinging the laryngoscope blade against the bridge was almost inevitable in this situation. Blade-tooth contact is extremely frequent in patients with reduced mouth opening or with Mallampati higher than II [5]. Oropharyngeal airways, such as Laryngeal masks, should also be used with caution in individuals with vulnerable anterior teeth or prostheses [6]. 

It is difficult to guess whether alternative devices for moderate-difficult intubation, such as video laryngoscopes (Glidescope, McGrath, Trueview, Trachlight, etc.) would have been less traumatic in this patient.

There is some evidence that using a mouthguard helps protect poor teeth during tracheal intubation, even though routine use of mouthguards has not proven useful and is not recommended on a regular basis [7].

In the case reported, the use of a protective “reinforced” toothpaste would not have reduced the possibility of detachment of the bridge since the simple yet light traction of the protective paste in the presence of an extreme fragility of the diseased roots could have caused the avulsion.

It could be speculated that more thorough scrutiny of the patient's upper front bridge during preanesthesia evaluation could have discovered the marked defects of the supporting native roots which predisposed the patient to irreparable damage during face mask ventilation and intubation [8]. This observation might have led to adequate information being imparted to the patient on the high risk of detachment during intubation. This patient was very likely unaware of his condition. Warning him of the possibility of a serious injury might have reduced his discontent once he realized the bridge had dislodged. 

The lack of appropriate documentation of the advanced periodontal disease in the anesthesia records likely reinforced the reimbursement claim. Even though the preexisting root and bridge deficits were well reported by the consulting dentist, it was not possible to downnegotiate the amount of compensation; the plaintiff particularly underlined the absence of anesthesiological documentation on the state of dental stumps, no mention of potential risks on anesthesia consent, and insufficient protective measures during airway instrumentation. 

In conclusion, dental lesions continue to be the most common cause of malpractice actions against anaesthesiologists, and claims mostly follow damage to teeth that have not been preoperatively identified as diseased. Even though the anesthesiologist cannot be held responsible for injuring teeth already damaged, ignoring or refusing to acknowledge responsibility for injured tooth or dental work may be a source of litigation if a patient is unaware of the existence of predisposing conditions. Documentation of the risk of dental lesion helps diminish the perception of medical negligence, while offering reimbursement in cases of malpractice or involuntary injury greatly decreases the likelihood that patient will pursue a claim.
